# Supplementary material for: Genome-Wide Identification and Expression Analysis of the Ginkgo biloba B-Box Gene Family in Response to Hormone Treatments, Flavonoid Levels, and Water Stress
Source: Int J Mol Sci. 2025 Aug 29;26(17):8427. doi: 10.3390/ijms26178427 (PMC12429349; doi:10.3390/ijms26178427)
Supplement: Supplementary file 1 [file ijms-26-08427-s001.zip › Supplementary files/Supplementary Figures.pptx]

## Slide 1
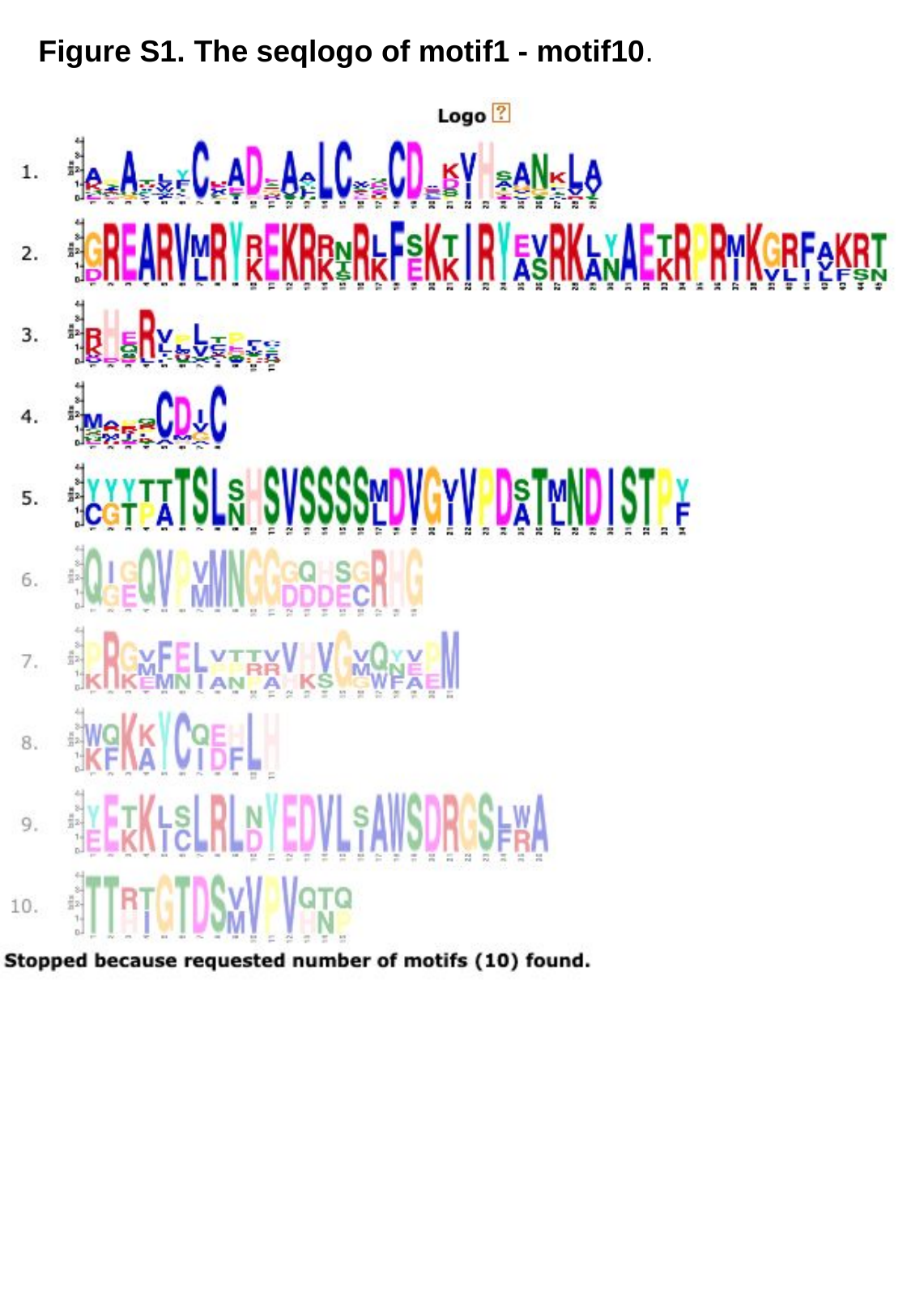

Figure S1. The seqlogo of motif1 - motif10.

## Slide 2
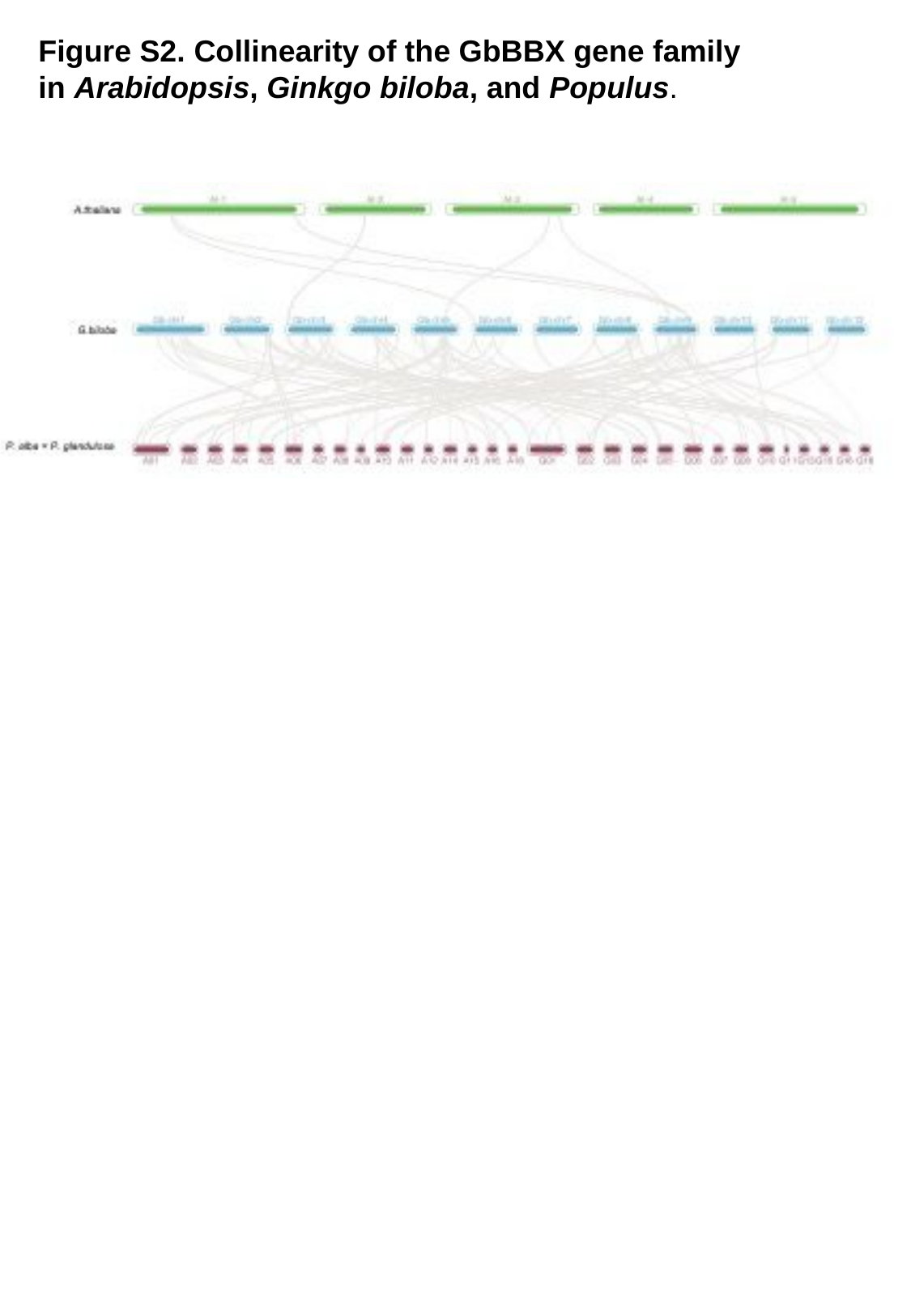

Figure S2. Collinearity of the GbBBX gene family in ﻿Arabidopsis, Ginkgo biloba, and Populus.
